# Real‐world multiple myeloma front‐line treatment and outcomes by transplant in the United States

**DOI:** 10.1002/jha2.739

**Published:** 2023-08-04

**Authors:** Joshua Richter, Darren Pan, Taylor Salinardi, Megan S. Rice

**Affiliations:** ^1^ Tisch Cancer Institute Icahn School of Medicine at Mount Sinai New York New York USA; ^2^ Sanofi Cambridge Massachusetts USA; ^3^ Present address: Azurity Pharmaceuticals Woburn Massachusetts USA; ^4^ Present address: Vertex Pharmaceuticals Cambridge Massachusetts USA

**Keywords:** multiple myeloma, real world, transplant

## Abstract

Stem cell transplantation (SCT) has been an integral treatment modality for multiple myeloma (MM) for decades. However, as standard‐of‐care therapies have improved, the benefit of SCT has been repeatedly called into question. This retrospective study evaluated the association between SCT in the first line of therapy (LOT) and outcomes for patients with newly diagnosed multiple myeloma (NDMM) in the United States. We included patients from a de‐identified electronic health record‐derived database who initiated front‐line MM therapy between January 1, 2016, and January 31, 2022. Overall, 18.8% (1127 of 5996 patients) received SCT in the first LOT. Multivariable‐adjusted Cox proportional hazards models, in which SCT was modeled as time varying, revealed longer real‐world progression‐free survival (rwPFS; hazard ratio [HR] 0.49; 95% confidence interval [CI] 0.43–0.57) and real‐world overall survival (rwOS; HR 0.47; 95% CI 0.39–0.56) for patients who received SCT in the first LOT. The degree of rwPFS and rwOS benefit imparted by SCT was consistent across all subgroups examined, including patients aged ≥75 years, women, non‐Hispanic Black/African American patients, those with renal impairment, and those with high‐risk cytogenetics. Findings from this analysis of real‐world patients with NDMM suggest that SCT remains an important standard of care in the era of novel therapies.

## Graphical abstract

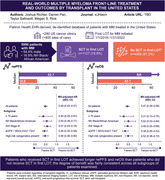

## 1 INTRODUCTION

Multiple myeloma (MM) is a hematologic malignancy of terminally differentiated plasma cells. Representing the second most common hematologic malignancy worldwide [1, 2], an estimated 34,470 new cases of MM and 12,640 deaths will occur from MM in the United States in 2022 [3].

Based on National Cancer Institute Surveillance, Epidemiology, and End Results data from 2012 to 2018, MM carries a 5‐year relative survival rate of 57.9% [3]. Although recent advances in therapy have improved outcomes for patients with MM, the disease remains incurable, and most patients continuously relapse over time [1, 4]. In the era of modern treatments, stem cell transplantation (SCT) remains the standard of care after primary therapy in transplant‐eligible patients [1, 4, 5], with a shift toward using functional status rather than a defined age cutoff to determine transplant eligibility [6].

Options for pre‐SCT induction as well as post‐SCT maintenance (with or without consolidation) continue to expand [1, 4, 5], but the lack of randomized head‐to‐head clinical trials prevents full understanding of the ideal sequence of therapies for patients with newly diagnosed MM (NDMM). Importantly, real‐world (RW) evidence can augment information learned from clinical trials and provide a greater understanding of treatment patterns and their impact on patient outcomes in everyday practice.

The aim of this study was to evaluate patient and disease characteristics, front‐line therapies, and patient outcomes, both overall and by transplant receipt in the first line of therapy (LOT), among RW patients with NDMM in the United States. Associations between baseline patient and disease characteristics and outcomes were also evaluated.

## 2 METHODS

### 2.1 Study design and data source

This retrospective, observational cohort study used the nationwide Flatiron Health electronic health record (EHR)‐derived, de‐identified database of patients with MM treated in the United States. The Flatiron Health database is a longitudinal database, comprising de‐identified patient‐level structured and unstructured data, curated via technology‐enabled abstraction [7, 8]. During the study period, the de‐identified data originated from approximately 280 US cancer clinics (∼800 sites of care).

The RW cohort used for this study included a probabilistic sample of US patients diagnosed with MM on or after January 1, 2011, who had at least two clinic encounters evident in the Flatiron Health database occurring after January 1, 2011, and until January 31, 2022. The index date was defined as the date of initiation of front‐line therapy. The baseline period was defined as the 60 days prior to and including the index date.

### 2.2 Patient selection

Patients with MM whose front‐line therapy was initiated between January 1, 2016, and January 31, 2022, were selected from the Flatiron Health MM cohort. Patients with a “line 0” treatment regimen (i.e., those with evidence of treatment from unstructured activity >30 days prior to the start of structured activity and those whose first MM treatment was SCT) were excluded, as were patients without evidence of structured activity within the 90 days following MM diagnosis. Patients without at least one LOT, with evidence of MM treatment more than 14 days prior to their diagnosis date, who participated in a clinical trial at any time, and/or with a second SCT as part of first LOT were also excluded.

### 2.3 Assessments and outcomes

Patient and disease characteristics, as well as hematologic abnormalities, were examined overall and by SCT receipt in line 1 of therapy (Supporting Information). High‐risk cytogenetics were defined as the presence of del(17p), t[4;14], and/or t[4;16]. Presence of 1q21+ was defined as gain (3 copies) or amplification (≥4 copies) of 1q21.

Front‐line treatment characteristics (e.g., primary therapy by treatment class) were examined overall and by SCT receipt (Supporting Information). Post‐SCT consolidation and/or maintenance therapy and treatment sequencing during line 1 were assessed only for patients who received SCT during line 1, all of whom had confirmed activity in the Flatiron Health database in the 180 days post‐SCT.

RW progression‐free survival (rwPFS) and RW overall survival (rwOS), by SCT receipt in line 1, were examined for the overall study population and, in post hoc analyses, for subgroups defined by age, sex, race/ethnicity, International Staging System (ISS) stage at diagnosis, cytogenetic risk (including individual examination of del(17p) and 1q21+), kidney function, and primary treatment during line 1. rwPFS was defined as the time from the start of front‐line therapy (i.e., the index date) to the date of first progression event (informed by International Myeloma Working Group criteria and incorporating both abstracted M‐spike values and structured free light chain values) or death. Progression events occurring within 30 days after the start of therapy were excluded, as such events are not expected to reflect progression on the therapy of interest. Patients without an observed date of progression or death were censored at the last lab date. As the timing and frequency of laboratory tests can vary (with less frequent testing leading to longer PFS), a sensitivity analysis was conducted to exclude patients with more than a 180‐day gap between the date of the progression event and the previous lab test. rwOS was defined as the time from the index date to the date of death. Patients without an observed date of death were censored at the last confirmed activity date (i.e., the later of the last visit date or the last date of selected unstructured activity).

RW time to treatment discontinuation (rwTTD) analyses were conducted for line 1 initial regimens (all patients) and line 1 post‐SCT maintenance therapy (among SCT patients only) (Supporting Information).

### 2.4 Statistical analysis

Patients were categorized as having received SCT in line 1 of therapy (SCT group) versus not receiving SCT in line 1 (no‐SCT group). Patient and disease characteristics at the start of first LOT, hematologic abnormalities, and treatment characteristics for line 1 were summarized descriptively using mean (standard deviation) and/or median (interquartile range) for continuous variables and frequencies and percentages for categorical variables.

Kaplan–Meier (KM) curves were constructed from the start of first LOT to progression or death (for rwPFS) and from the start of first LOT to death (for rwOS), with the median (95% confidence interval [CI]) reported for each outcome.

Differences in rwPFS and rwOS by SCT in line 1 of therapy, overall and by subgroups, were assessed using multivariable (MV)‐adjusted Cox proportional hazards models, in which SCT was modeled as a time‐varying covariate. Models were adjusted for age at the start of first LOT (<65, 65 to <75, ≥75 years), sex (female, male), race/ethnicity (non‐Hispanic White, non‐Hispanic Black, Hispanic or Latinx, other, missing), practice type (community, academic), M‐protein subtype at diagnosis (IgG, IgA, light chain, other, missing), ISS stage at diagnosis (I, II, III, missing), Eastern Cooperative Oncology Group (ECOG) performance status (PS) at the start of first LOT (0, 1, ≥2, missing), cytogenetic risk (high/standard), 1q21+ (present, absent), estimated glomerular filtration rate at start of the LOT (<60, ≥60 mL/min/1.73 m^2^, missing), and time from diagnosis to start of line 1 (continuous). The KM method was used to analyze rwTTD, overall and by SCT (Supporting Information). All statistical analyses were performed using R.

## 3 RESULTS

### 3.1 Patient demographics and clinical characteristics

A total of 5996 patients with MM were included in the study cohort; 1127 (18.8%) patients received SCT in line 1 of therapy and 4869 (81.2%) patients did not (Table 1 and Figure S1). From year to year, the proportion of patients receiving SCT as part of their first‐line treatment remained consistent (annual range 17.2%–20.1% for patients initiating frontline therapy between 2016 and 2020). For the overall population, the median age at diagnosis and start of line 1 of therapy was 70 years, 54.4% of patients were male, 55.8% were non‐Hispanic White, 16.9% were non‐Hispanic Black/African American, and 6.7% were Hispanic or Latinx. Patients who received SCT were younger at diagnosis and start of first LOT (63 years vs. 72 years for both comparisons), had lower ISS disease stage at diagnosis, and were more likely to be treated in academic versus community settings than those who did not receive SCT. Non‐Hispanic White and Hispanic or Latinx patients were slightly more likely than non‐Hispanic Black/African American patients to receive SCT (21.3% and 20.5% vs. 16.5%, respectively). SCT was less likely among patients with renal impairment (defined as estimated glomerular filtration rate <60 mL/min/1.73 m^2^) and poorer ECOG PS at start of front‐line therapy. Rates of hematologic abnormalities (i.e., values less than the lower limit of normal) derived from structured laboratory data did not differ between the SCT and no‐SCT groups (Table S1).

**TABLE 1:** Baseline patient and disease characteristics, overall and by stem cell transplant (SCT) receipt in line 1 of therapy.

Characteristic	Overall (*n* = 5996)	SCT group (*n* = 1127)	No‐SCT group (*n* = 4869)
Age at MM diagnosis (years), median (IQR)	70 (62–77)	63 (57–69)	72 (64–79)
Age at MM diagnosis (categorical years)			
<65	1869 (31.2)	617 (54.8)	1252 (25.7)
65 to <75	2081 (34.7)	471 (41.8)	1610 (33.1)
≥75	2046 (34.1)	39 (3.5)	2007 (41.2)
Age at start of LOT (years), median (IQR)	70 (62–77)	63 (57–69)	72 (64–79)
Age at start of LOT (categorical years)			
<65	1853 (30.9)	613 (54.4)	1240 (25.5)
65 to <75	2082 (34.7)	474 (42.1)	1608 (33.0)
≥75	2061 (34.4)	40 (3.6)	2021 (41.5)
Sex			
Female	2734 (45.6)	496 (44.0)	2238 (46.0)
Male	3262 (54.4)	631 (56.0)	2631 (54.0)
Race			
Non‐Hispanic Black/African American	1014 (16.9)	167 (14.8)	847 (17.4)
Non‐Hispanic White	3347 (55.8)	712 (63.2)	2635 (54.1)
Hispanic or Latinx	404 (6.7)	83 (7.4)	321 (6.6)
Other	612 (10.2)	77 (6.8)	535 (11.0)
Missing	619 (10.3)	88 (7.8)	531 (10.9)
Region of residence			
Northeast	961 (16.0)	172 (15.3)	789 (16.2)
Midwest	748 (12.5)	161 (14.3)	587 (12.1)
South	2416 (40.3)	431 (38.2)	1985 (40.8)
West	901 (15.0)	132 (11.7)	769 (15.8)
Other/missing	970 (16.2)	231 (20.5)	739 (15.2)
Practice type			
Academic	730 (12.2)	194 (17.2)	536 (11.0)
Community	5189 (86.5)	914 (81.1)	4275 (87.8)
M‐protein type			
IgG	3263 (54.4)	671 (59.5)	2592 (53.2)
IgA	1211 (20.2)	242 (21.5)	969 (19.9)
Light chain	1208 (20.2)	183 (16.2)	1025 (21.1)
Other	65 (1.1)	18 (1.6)	47 (1.0)
Missing	249 (4.2)	13 (1.2)	236 (4.9)
ISS stage at diagnosis			
Stage I	1247 (20.8)	370 (32.8)	877 (18.0)
Stage II	1209 (20.2)	257 (22.8)	952 (19.6)
Stage III	1252 (20.9)	207 (18.4)	1045 (21.5)
Missing	2288 (38.2)	293 (26.0)	1995 (41.0)
Cytogenetic risk (assessed at any time)			
High risk^a^	947 (15.8)	235 (20.9)	712 (14.6)
Standard risk	1713 (28.6)	298 (26.4)	1415 (29.1)
Missing	3336 (55.6)	594 (52.7)	2742 (56.3)
1q21+^b^ (assessed at any time)			
Present	1256 (21.0)	264 (23.4)	992 (20.4)
Absent	2120 (35.4)	422 (37.4)	1698 (34.9)
Missing	2620 (43.7)	441 (39.1)	2179 (44.8)
eGFR^c^ (mL/min/1.73 m^2^) at start of LOT			
<60	2057 (34.3)	274 (24.3)	1783 (36.6)
≥60	2343 (39.1)	561 (49.8)	1782 (36.6)
Missing	1596 (26.6)	292 (25.9)	1304 (26.8)
ECOG PS at start of LOT			
0	1448 (24.2)	328 (29.1)	1120 (23.0)
1	1536 (25.6)	274 (24.3)	1262 (25.9)
≥2	841 (14.0)	85 (7.5)	756 (15.5)
Missing	2171 (36.2)	440 (39.0)	1731 (35.6)
Year of LOT start			
2016	1026 (17.1)	203 (18.0)	823 (16.9)
2017	990 (16.5)	215 (19.1)	775 (15.9)
2018	1065 (17.8)	227 (20.1)	838 (17.2)
2019	1061 (17.7)	219 (19.4)	842 (17.3)
2020	955 (15.9)	194 (17.2)	761 (15.6)
2021	899 (15.0)	69 (6.1)	830 (17.1)
Time from MM diagnosis to LOT start (months), median (IQR)	1.06 (0.65–1.52)	1.06 (0.68–1.48)	1.03 (0.65–1.52)

*Note*: Data are *n* (%) unless otherwise noted.

Abbreviations: ECOG, Eastern Cooperative Oncology Group; eGFR, estimated glomerular filtration rate; Ig, immunoglobulin; IQR, interquartile range; ISS, International Staging System; LOT, line of therapy; MM, multiple myeloma; PS, performance status.

^a^ High‐risk cytogenetics were defined as the presence of ≥1 of del(17p), t(4;14), or t(14;16).

^b^ 1q21+ was defined as gain (3 copies) or amplification (≥4 copies) of 1q21.

^c^ Assessed using the Modification of Diet in Renal Disease (MDRD) equation.

### 3.2 Treatment patterns

Initial therapy, overall and by SCT receipt in line 1 of therapy, is shown in Table 2. The most frequently prescribed initial therapy among patients in the SCT and no‐SCT groups was a proteasome inhibitor (PI) + immunomodulatory drug (IMiD)‐based regimen (in 72.2% and 49.0% of patients, respectively). PI‐based and monoclonal antibody (MAb)‐based initial therapies were more common in the no‐SCT group than in the SCT group (15.8% vs. 1.3% and 7.8% vs. 3.8%, respectively).

**TABLE 2:** Initial therapy, overall and by SCT receipt in line 1.

Initial therapy	Overall (*N* = 5996)	SCT group (*n* = 1127)	No‐SCT group (*n* = 4869)
PI based^a^	786 (13.1)	15 (1.3)	771 (15.8)
IMiD based^a^	715 (11.9)	120 (10.7)	595 (12.2)
PI + IMiD based^a^	3202 (53.4)	814 (72.2)	2388 (49.0)
Chemotherapy based^b^	870 (14.5)	135 (12.0)	735 (15.1)
MAb based^c^	421 (7.0)	43 (3.8)	378 (7.8)
Other^d^	<5 (<0.1)	<5 (<0.4)	<5 (<0.1)

*Note*: Data are *n* (%).

Abbreviations: BCMA, B‐cell maturation antigen; IMiD, immunomodulatory drug; MAb, monoclonal antibody; PI, proteasome inhibitor; SCT, stem cell transplant.

^a^ Regimens containing only the drug class(es) listed (±steroids).

^b^ Regimens containing at least one chemotherapy agent (±steroids) that could also include PIs and IMiDs but not MAb or “other” drugs (see footnote “d”).

^c^ Regimens containing at least one MAb agent (±steroids) that could also include PIs, IMiDs, and chemotherapy agents but not “other” drugs (see footnote “d”).

^d^ Included BCMA‐targeting drugs (e.g., belantamab mafodotin, idecabtagene vicleucel) or those with novel mechanisms of action (e.g., panobinostat, selinexor, melflufen).

Bortezomib/lenalidomide/dexamethasone was the most common primary treatment in both the SCT group (64.8%) and no‐SCT group (45.5%) (Table S2). In the SCT group, 64.1% of patients received post‐SCT maintenance therapy and 7.1% received post‐SCT consolidation therapy. Patient and disease characteristics for the SCT group, by post‐SCT maintenance and consolidation therapy receipt, are shown in Table S3. Overall, the most common line 1 treatment sequence in the SCT group was PI + IMiD‐based therapy, followed by SCT, then maintenance IMiD (Table S4).

### 3.3 Outcomes

**FIGURE 1:**
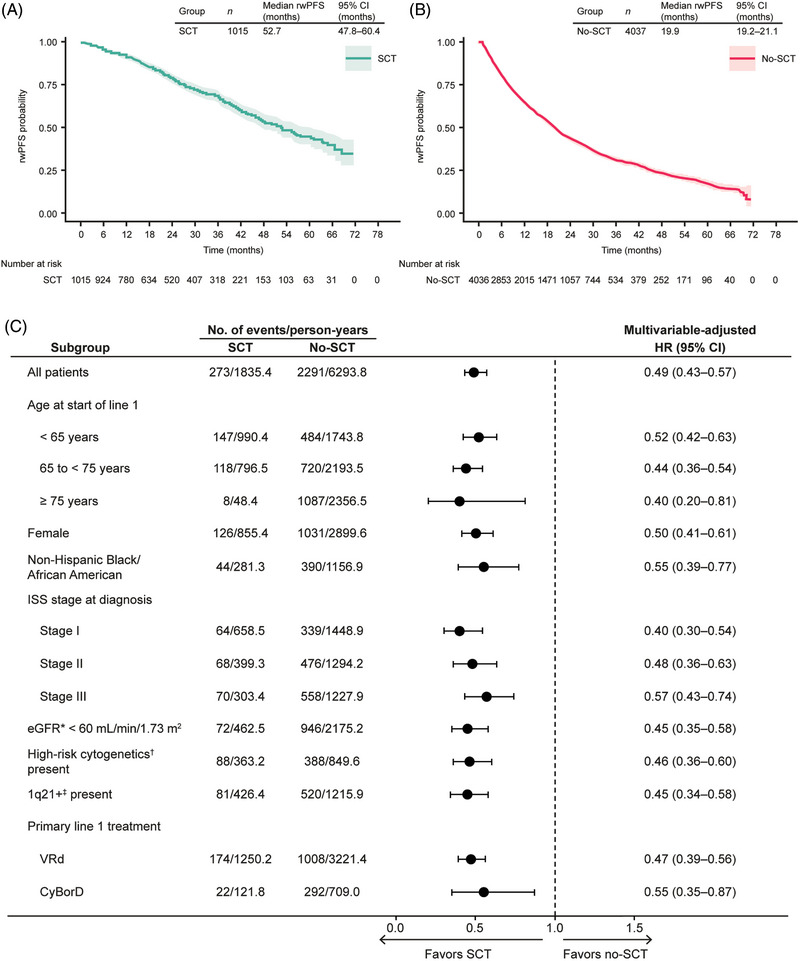
Real‐world progression‐free survival (PFS) from start of initial therapy (A) for the overall stem cell transplant (SCT) group, (B) for the overall no‐SCT group, and (C) for select subgroups, by SCT receipt in line 1. ^*^Assessed using the Modification of Diet in Renal Disease (MDRD) equation. ^†^High‐risk cytogenetics were defined as the presence of ≥1 of del(17p), t(4;14), or t(14;16). ^‡^1q21+ was defined as gain (3 copies) or amplification (≥4 copies) of 1q21. CI, confidence interval; CyBorD, cyclophosphamide/bortezomib/dexamethasone; eGFR, estimated glomerular filtration rate; HR, hazard ratio; ISS, International Staging System; rwPFS, real‐world progression‐free survival; VRd, bortezomib/lenalidomide/dexamethasone.

The rwPFS population consisted of 5052 patients (Figure S1), whereas rwOS and rwTTD analyses were performed for the full study population (*N* = 5996). KM analysis revealed a median time to RW progression of 52.7 months (95% CI 47.8–60.4) versus 19.9 months (95% CI 19.2–21.1) from the start of first LOT in the SCT versus no‐SCT groups, respectively (Figure 1A,B). In the Cox proportional hazard model, patients who received SCT in line 1 had longer rwPFS than those who did not (MV‐adjusted hazard ratio [HR] 0.49; 95% CI 0.43–0.57; Figure 1C). rwPFS KM and MV‐adjusted Cox model findings were confirmed by sensitivity analyses that excluded patients with more than a 180‐day gap between progression event and previous laboratory test.

**FIGURE 2:**
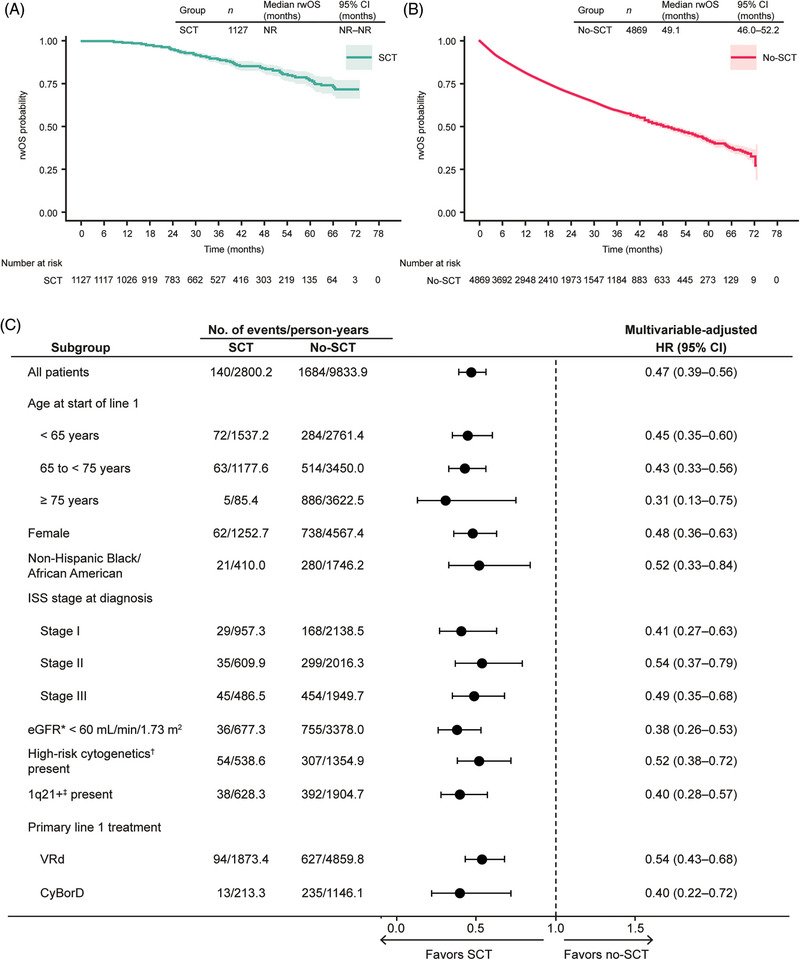
Real‐world overall survival (OS) from start of initial therapy (A) for the overall stem cell transplant (SCT) group, (B) for the overall no‐SCT group, and (C) for select subgroups, by SCT receipt in line 1. ^*^Assessed using the Modification of Diet in Renal Disease (MDRD) equation. ^†^High‐risk cytogenetics were defined as the presence of ≥1 of del(17p), t(4;14), or t(14;16). ^‡^1q21+ was defined as gain (3 copies) or amplification (≥4 copies) of 1q21. CI, confidence interval; CyBorD, cyclophosphamide/bortezomib/dexamethasone; eGFR, estimated glomerular filtration rate; HR, hazard ratio; ISS, International Staging System; NR, not reached; rwOS, real‐world overall survival; VRd, bortezomib/lenalidomide/dexamethasone.

In KM analysis for rwOS, median time to death was not reached (95% CI not reached–not reached) versus 49.1 months (95% CI 46.0–52.2) from the start of first LOT in the SCT versus no‐SCT groups, respectively (Figure 2A,B). On average, each person contributed 2.1 years of observation to the rwOS analyses. In the Cox proportional hazard model, patients who received SCT in line 1 had longer rwOS than those who did not (MV‐adjusted HR 0.47; 95% CI 0.39–0.56; Figure 2C). MV‐adjusted HRs for rwPFS (Figure 1C and Table S5) and rwOS (Figure 2C and Table S6) were fairly consistent across all subgroups examined, ranging from 0.38 to 0.57 for rwPFS and from 0.28 to 0.64 for rwOS.

The median rwTTD for the line 1 initial therapy regimen was 4.2 months (95% CI 4.1–4.4) in the SCT group and 6.5 months (95% CI 6.2–6.8) in the no‐SCT group. Notably, the longer duration seen in the no‐SCT group may represent some capturing of maintenance therapy as initial therapy. Among the 722 patients who received post‐SCT maintenance, the median rwTTD for the line 1 post‐SCT maintenance regimen was 28.2 months (95% CI 24.8–33.7).

## 4 DISCUSSION

This study utilized recent EHR‐derived data to retrospectively analyze RW treatment characteristics and patient outcomes, both overall and by SCT receipt in line 1 of therapy, to complement learnings from prospective clinical trials that may not be fully generalizable to the NDMM population in the United States. To our knowledge, this study is the first to analyze rwPFS and rwOS benefits, by first‐line SCT receipt, among numerous subgroups of patients.

Initial therapy with a PI + IMiD‐based regimen was most common across the overall study population, both for patients who did and did not receive SCT during line 1 of therapy. This is consistent with findings from other US‐based RW studies utilizing slightly earlier data sets from Flatiron Health [9, 10]. Notably, use of PI + IMiD‐based initial therapy in our study was higher among the SCT versus no‐SCT group (72.2% vs. 49.0%, respectively), whereas PI‐based initial therapy was more common in the no‐SCT group (15.8% vs. 1.3% in the SCT group). This likely reflects a tendency to treat the older no‐SCT group with gentler induction regimens and is consistent with findings from earlier US‐based RW studies that showed similar treatment patterns in SCT versus no‐SCT populations [11] and in transplant‐eligible versus transplant‐ineligible patients [9].

Previous analyses of front‐line MM prescribing trends showed that rates of MAb‐based initial therapies (including quadruplet regimens) were no higher than 3% through 2019 [9, 12]. The use of MAb‐based initial therapies was 7% in our overall study population, which suggests increased uptake of such regimens in the United States between 2019 and early 2022. Results from ALCYONE (daratumumab–bortezomib–melphalan–prednisone), MAIA (daratumumab–lenalidomide–prednisone), and GRIFFIN (daratumumab–lenalidomide–bortezomib–prednisone) were first reported in 2018, 2019, and 2020, respectively [13, 14, 15]. Similarly, results from GMMG‐HD7 (isatuximab–lenalidomide–bortezomib–prednisone) were first reported (in abstract form) in 2021 and were published in late 2022 [16, 17]. Thus, the time period of our study (January 1, 2016, to January 31, 2022) does not fully reflect the emergence of anti‐CD38 MAbs into the NDMM setting and may underestimate their current representation among front‐line therapies. Ongoing clinical trials of anti‐CD38‐based therapies in SCT‐eligible patients (e.g., PERSEUS [18]) and among patients not intended for early SCT (e.g., IMROZ [19], EQUATE [20], and CEPHEUS [21]) may lead to further uptake of such regimens in everyday practice. As such, regular reassessment of RW data will be essential for identifying the impact of anti‐CD38‐based triplet and quadruplet therapies on patient outcomes, both overall and when used alongside SCT in front‐line therapy.

Roughly one in five patients in our study received SCT as part of line 1 of therapy. This remained relatively consistent among patients starting front‐line therapy during any year between 2016 and 2020, showing that SCT remains an essential part of NDMM treatment in the age of novel therapies. RW use of SCT, specifically as part of the first LOT, has been infrequently examined. A National Cancer Database Analysis utilizing data from 1998 to 2010 reported that 9% of patients received SCT as part of front‐line therapy [22]. This is substantially lower than the 18.8% of patients in our study who received SCT in the first line, suggesting greater use of front‐line SCT in the United States over the last decade. Increasing use of early SCT for treatment of NDMM is supported by consistent findings of improved PFS with the inclusion of SCT as part of front‐line therapy in prospective clinical trials [23, 24, 25, 26] and, in RW analyses, of PFS benefit when SCT is received early (<12 months after diagnosis) rather than late (≥12 months after diagnosis) [27]. However, reports of OS benefit have been somewhat inconsistent in the literature [23–26, 28–31], with benefit sometimes only emerging after longer‐term follow‐up [32].

Although RW endpoints inherently differ from endpoints used in clinical trials, the use of rwPFS as a meaningful outcome is increasing [27, 33, 34]. Utilizing Flatiron Health's rules for PFS and MV‐adjusted Cox proportional hazards models that modeled SCT as a time‐varying covariate, our study showed that patients who received SCT in line 1 achieved longer rwPFS than patients who did not receive SCT in line 1. This benefit was fairly consistent across all subgroups examined, including patients aged 75 years or older, women, non‐Hispanic Black/African American patients, those with ISS stage II or III disease at diagnosis, those with renal impairment, and those with high‐risk cytogenetics and/or 1q21+.

The association we found between front‐line use of SCT and improved rwPFS aligns well with results from the recently published DETERMINATION trial [26], which compared outcomes among transplant‐eligible patients with NDMM who were treated with either lenalidomide–bortezomib–dexamethasone alone or in combination with early SCT (followed by lenalidomide maintenance therapy until disease progression in both groups). Early ASCT led to improved PFS; the lenalidomide–bortezomib–dexamethasone alone group had a 53% higher risk of disease progression or death than the SCT group (HR 1.53; 95% CI 1.23–1.91; *p* < 0.001). This association remained generally consistent among subgroups examined, although authors noted that their trial was not powered to evaluate PFS in subgroups [26]. The rwPFS benefit in our study is also consistent with results from the IFM2009 study, which showed that age, sex, ISS disease stage, and cytogenetic risk profile did not significantly alter the PFS benefit achieved with the addition of early SCT to lenalidomide–bortezomib–dexamethasone versus lenalidomide–bortezomib–dexamethasone alone [23], and with results from the EMN02/H095 trial, which revealed PFS superiority of SCT over bortezomib/melphalan/prednisone in patients with ISS disease stage 2 or 3 and those with high‐risk cytogenetics [24].

Our study also showed longer rwOS among patients who received SCT in line 1 compared with those who did not receive SCT in line 1. This is consistent with a recent RW analysis from Canada that examined a cohort of patients with NDMM who initiated first‐line therapy between 2007 and 2018 [29]. The outcome of OS was stratified by upfront SCT, revealing a median OS of 122.0 months versus 54.3 months from first‐line treatment in the SCT versus no‐SCT groups, respectively. Whereas 86.5% of patients in our study were treated at community‐based clinics in the United States, the RW data utilized in the Canadian study originated from 15 academic sites, most located in urban areas and affiliated with university teaching hospitals. During first LOT, use of lenalidomide and anti‐CD38 MAbs during first LOT was substantially lower in the Canadian cohort and cyclophosphamide use was substantially higher, which the authors partially attributed to the lack of public funding of novel agents in the Canadian healthcare system. Despite these key differences between our study and the Canadian study, the survival benefit seen with SCT is consistent, suggesting that our results may be quite generalizable across different RW populations within the United States and other countries, perhaps including populations with poorer access to novel therapeutic agents.

The association we found between front‐line SCT use and rwOS benefit was not observed in the IFM 2009 [23] and DETERMINATION [26] trials. Patient populations were different, which may have contributed to a wider distinction in survival outcomes between the SCT and no‐SCT groups in our study. The median age of our overall study cohort was 70 years, whereas the median ages of the IFM 2009 and DETERMINATION study populations were approximately 59.5 and 56 years, respectively. Of the two US‐based trials, over 86% of patients in our study were treated at community‐based practices, whereas the DETERMINATION study was conducted predominantly in academic centers. In addition, roughly one‐quarter of patients who did not receive SCT as part of study treatment in the DETERMINATION trial went on to receive SCT in subsequent lines of therapy [26], which may have made OS results more similar between treatment arms. DETERMINATION trial authors proposed that the lack of OS benefit in their early SCT group was at least partly due to the multiple highly efficacious treatment options that had become available in the second line and beyond over the last decade. Our study population received a variety of front‐line therapies rather than the initial therapy mandated in the DETERMINATION trial (lenalidomide–bortezomib–dexamethasone). In fact, only 49.1% of patients in our study received lenalidomide–bortezomib–dexamethasone as their primary treatment. Additional studies of RW patients with NDMM, particularly those receiving triplet therapies as first LOT, are needed to fully examine the rwOS benefit of front‐line SCT moving forward in the era of novel therapies.

As mentioned previously, our RW population included patients of more advanced age (69.1% were ≥65 years old and 34.4% were ≥75 years old at start of first LOT) than the IFM2009, EMN02/H095, and DETERMINATION trials, which capped enrollment at 65 years [23, 24, 26]. The overall and SCT cohorts in our study were also older than those examined in the Canadian RW study (median age of 70 years vs. 64 years for the overall populations and 63 years vs. 59 years for the SCT populations, respectively) [29]. Despite this, rwPFS and rwOS benefits of SCT were still evident among the overall population in our study. Notably, the cohorts of patients aged 75 years or older who received SCT in line 1 of therapy remained small (39 patients), as did the cohort of patients with ECOG PS of 2 or higher (85 patients). Thus, the true effect of early SCT in elderly and/or more frail patients may not be fully represented by our study results.

Our study had some limitations. The Flatiron Health MM Cohort comprises predominantly patients treated at community‐based centers. As such, the resulting study cohorts may not be as representative of patients treated at large academic centers. However, non‐Hispanic Blacks/African Americans and Hispanic or Latinx patients comprised 16.9% and 6.7% of the overall study population, suggesting appropriate representation of these races/ethnicities based on disease epidemiology and its estimated incidence in non‐White patients [35, 36]. Black/African American patients comprised 20% of the DETERMINATION trial population; in that trial, Black/African American patients did not see the same PFS benefit from front‐line SCT as White/Caucasian patients (PFS HR of 1.07 [95% CI 0.61–1.89] vs. PFS HR of 1.67 [95% CI 1.29–2.15], respectively) [26]. In addition, front‐line SCT did not appear to result in any differential benefit in OS between races (HRs for death were similar between races and did not reach statistical significance in either group) [26]. These findings from the DETERMINATION trial are discrepant with RW findings that Black patients with MM have better overall survival than White patients when treated equally [37, 38]. Our study revealed rwPFS and rwOS benefits of front‐line SCT among both non‐Hispanic White and non‐Hispanic Black/African American patients, but we did not specifically examine differential benefit between races/ethnicities. A better understanding of racial/ethnic differences that may exist in both biology of disease (e.g., cytogenetics) and baseline characteristics (e.g., renal function), and to what extent they may impact treatment choices and survival outcomes, is needed.

Because patients who received SCT had to survive long enough to get SCT, the KM analyses were subject to immortal person‐time among the SCT group, which could have biased outcomes in their favor. Importantly, patients who did not receive SCT during front‐line treatment in our study represent a combination of those deemed transplant‐ineligible, those considered eligible for SCT who died or left the Flatiron Health system prior to SCT, or those who were otherwise lost to follow‐up. Therefore, the results for non‐SCT patients in our study may have limited generalizability to transplant‐ineligible patients. Notably, transplant ineligibility is difficult to derive from EHR data, as evidenced by the use of surrogates such as age in the RW literature [9].

As with most EHR‐based studies, the Flatiron Health database may also not capture all treatment received by included patients. To account for this, patients whose data suggested potential missing treatment (e.g., those with evidence of treatment from unstructured activity >30 days prior to the start of their structured activity in the EHR) were excluded, and time‐to‐event analyses censored patients to reduce the influence of loss to follow‐up. To account for potential bias due to confounding, which is inherent to all observational studies, Cox proportional hazard models were adjusted for several baseline factors, although we may not have adjusted for all differences between the SCT and no‐SCT groups. In addition, because less frequent laboratory testing can contribute to extended PFS findings, a sensitivity analysis was conducted to exclude patients with more than a 180‐day gap between the date of their progression event and their previous laboratory test. The results of the sensitivity analysis were similar to those of the primary analysis.

Using information derived from an RW database, we provide a robust analysis of NDMM treatment patterns and outcomes according to SCT receipt in front‐line therapy across the United States, revealing that SCT remains an important standard of care in the era of novel therapies. Given the rapidly expanding treatment landscape and evolving definition of SCT eligibility, periodic re‐examination of RW data will be necessary to capture the true impact of novel treatment approaches, including MAb‐based triplet and quadruplet therapies, for patients with NDMM treated in everyday practice. Strategies to improve the abstraction of reliable data from EHRs as well as the accuracy of their statistical interpretation will help to strengthen the ability of RW data to augment learnings from clinical trials. Efforts should be made to improve representation of elderly patients, those with renal impairment, and those of non‐White race/ethnicity in clinical trials investigating the benefit of SCT.

## AUTHOR CONTRIBUTIONS

All authors were involved in the conception and design of the study, data interpretation, and manuscript writing. Megan S. Rice was responsible for data analysis. All authors participated in manuscript writing, review, and final approval of the submitted version of the manuscript.

## CONFLICT OF INTEREST STATEMENT

Joshua Richter: Janssen, BMS, Sanofi—speakers Bureau. Celgene, Janssen, BMS, Karyopharm, Sanofi, Takeda—advisory boards. BMS, Sanofi, Takeda—consulting. Tisch Cancer Institute, Icahn School of Medicine at Mount Sinai—current employment. Darren Pan: Tisch Cancer Institute, Icahn School of Medicine at Mount Sinai—current employment. Taylor Salinardi and Megan S. Rice: Sanofi—employment at the time of the study, may hold stock and/or stock options in the company.

## ETHICS STATEMENT

Not applicable.

## PATIENT CONSENT STATEMENT

Not applicable.

## PERMISSION TO REPRODUCE MATERIAL FROM OTHER SOURCES

Not applicable.

## CLINICAL TRIAL REGISTRATION

Not applicable.

## Supporting information

Supporting Information

## Data Availability

The de‐identified data that support the findings of this study are subject to a license agreement with Flatiron Health; interested researchers should contact DataAccess@flatiron.com to determine licensing terms.
