# Supplementary material for: Real‐world multiple myeloma front‐line treatment and outcomes by transplant in the United States
Source: EJHaem. 2023 Aug 4;4(4):984–94. doi: 10.1002/jha2.739 (PMC10660406; doi:10.1002/jha2.739)

**Real-World Multiple Myeloma Front-line Treatment and Outcomes by Transplant in the United States**

Joshua Richter, Darren Pan, Taylor Salinardi, Megan Rice

**SUPPLEMENT**

**SUPPLEMENTARY METHODS:**

**Assessments and outcomes**

***Patient and disease characteristics***

Estimated glomerular filtration rate (eGFR, using the Modification of Diet in Renal Disease [MDRD] equation) at the start of front-line therapy was calculated from creatinine lab values using the following equation: 175 × (creatinine mg/dL)-1.154 × (age at time of creatinine lab)-0.203 × (0.742 if female) × (1.212 if Black/African American). Extreme creatinine values (>99.99 percentile or <0.01 percentile) were set to missing. The creatinine value used to calculate eGFR was the closest non-missing value to the index date (within 60 days prior to and including the index date). If multiple creatinine values were available on the same day, they were averaged. Patients with missing race were categorized as missing eGFR. Eastern Cooperative Oncology Group performance status (ECOG PS) at the start of front-line therapy was determined by the closest non-missing ECOG PS value to the index date within the 60 days prior to and including the index date.

***Hematologic abnormalities***

The lowest laboratory value at baseline and during the initial treatment period for the overall population and by SCT status (Supplementary Table 1) were reported as follows:

- Anemia (Hemoglobin ≥lower limit of normal [LLN], <LLN, missing)
- Lymphocyte count decreased (Lymphocytopenia) (≥LLN, <LLN, missing)
- Platelet count decreased (Thrombocytopenia) (≥LLN, <LLN, missing)
- Neutrophil count decreased (Neutropenia) (≥LLN, <LLN, missing)
- White blood cell count decreased (Leukopenia) (≥LLN, <LLN, missing)

***Front-line treatment characteristics***

The following definitions were used to define drug groupings:

- Proteasome inhibitors (PIs): bortezomib, carfilzomib, ixazomib
- Immunomodulatory drugs (IMiDs): lenalidomide, pomalidomide, thalidomide
- Monoclonal antibody (MAb) agents: daratumumab, daratumumab/hyaluronidase-fihj, elotuzumab, isatuximab-irfc
- Chemotherapy: bendamustine, cisplatin, cyclophosphamide, doxorubicin, etoposide, liposomal doxorubicin, melphalan, vincristine
- Steroids: dexamethasone, prednisone
- “Other”:
  - B-cell maturation antigen-targeting drugs: belantamab mafodotin, idecabtagene vicleucel
  - Novel mechanism of action drugs: panobinostat, selinexor, melphalan flufenamide

For characterization of types of regimens, the following definitions were used:

- PI-based: regimens containing at least one PI (+/- steroids) but do not contain IMiDs, chemotherapies, MAbs, or “other” drugs (see above)
- IMiD-based: Regimens containing at least one IMiD (+/- steroids), but do not contain PIs, chemotherapies, MAbs, or “other” drugs (see above)
- PI+IMiD based: Regimens containing at least one IMiD and at least one PI (+/- steroids), but do not contain chemotherapies, MAbs, or “other” drugs (see above)
- Chemo-based: Regimens containing at least one chemotherapy agent (+/- steroids), but do not contain MAbs or “other” drugs (see above)
- Antibody-based: Regimens containing at least one MAb agent (+/- steroids), but do not contain “other” drugs (see above)
- Other: Regimens containing “other” treatments (see above)

***Real-world time to treatment discontinuation (rwTTD)***

For calculation of rwTTD, patients were observed from the first drug episode for that treatment to the last drug episode for that treatment. A patient was treated as uncensored if: a) the patient advanced to a new line of therapy (LOT)/LOT component (e.g., maintenance), b) the patient did not advance to a new LOT/LOT component but had a recorded date of death, c) the patient had not advanced to a new LOT/LOT component and had no recorded date of death, but had 120 days of confirmed structured activity after the last drug episode.

**Supplementary Table S1**. Most abnormal laboratory value during initial treatment period, by SCT receipt in line 1 of therapy

|  | **Patient population** | **Most abnormal lab value during initial treatment period, *n* (%)** | | |
| --- | --- | --- | --- | --- |
|  |  | **≥LLN** | **<LLN** | **Missing** |
| **Hemoglobin** | Overall (*N* = 5996) | 490 (8.17) | 4966 (82.82) | 540 (9.01) |
|  | SCT (*N* = 1127) | 138 (12.24) | 895 (79.41) | 94 (8.34) |
|  | No SCT (*N* = 4869) | 352 (7.23) | 4071 (83.61) | 446 (9.16) |
| **Lymphocyte count** | Overall (*N* = 5996) | 1430 (23.85) | 3599 (60.02) | 967 (16.13) |
|  | SCT (*N* = 1127) | 244 (21.65) | 700 (62.11) | 183 (16.24) |
|  | No SCT (*N* = 4869) | 1186 (24.36) | 2899 (59.54) | 784 (16.1) |
| **Neutrophil count** | Overall (*N* = 5996) | 2811 (46.88) | 1598 (26.65) | 1587 (26.47) |
|  | SCT (*N* = 1127) | 557 (49.42) | 246 (21.83) | 324 (28.75) |
|  | No SCT (*N* = 4869) | 2254 (46.29) | 1352 (27.77) | 1263 (25.94) |
| **White blood cell count** | Overall (*N* = 5996) | 2159 (36.01) | 3071 (51.22) | 766 (12.78) |
|  | SCT (*N* = 1127) | 411 (36.47) | 582 (51.64) | 134 (11.89) |
|  | No SCT (*N* = 4869) | 1748 (35.9) | 2489 (51.12) | 632 (12.98) |
| **Platelet count** | Overall (*N* = 5996) | 1940 (32.35) | 2973 (49.58) | 1083 (18.06) |
|  | SCT (*N* = 1127) | 385 (34.16) | 561 (49.78) | 181 (16.06) |
|  | No SCT (*N* = 4869) | 1555 (31.94) | 2412 (49.54) | 902 (18.53) |

Abbreviations: LLN, lower limit of normal; SCT, stem-cell transplant

**Supplementary Table S2.** Top primary treatment regimens, overall and by SCT receipt in line 1 of therapy

| **Primary treatment by agent, *n* (%)** | **Regimen type** | **Overall  (*N* *=* 5996)** | **SCT group (*N* *=* 1127)** | **No-SCT group (*N* *=* 4869)** |
| --- | --- | --- | --- | --- |
| bortezomib+dexamethasone+lenalidomide | PI + IMiD-based | 2944 (49.1) | 730 (64.8) | 2214 (45.5) |
| bortezomib+dexamethasone | PI-based | 763 (12.7) | 15 (1.3) | 748 (15.4) |
| dexamethasone+lenalidomide | IMiD-based | 673 (11.2) | 117 (10.4) | 556 (11.4) |
| bortezomib+cyclophosphamide+dexamethasone | Chemotherapy-based | 628 (10.5) | 76 (6.7) | 552 (11.3) |
| bortezomib+daratumumab+ dexamethasone+lenalidomide | MAb-based | 155 (2.6) | 31 (2.8) | 124 (2.5) |
| carfilzomib+dexamethasone+lenalidomide | PI + IMiD-based | 131 (2.2) | 63 (5.6) | 68 (1.4) |
| bortezomib+cyclophosphamide+ dexamethasone+lenalidomide | Chemotherapy-based | 100 (1.7) | 33 (2.9) | 67 (1.4) |
| daratumumab+dexamethasone+lenalidomide | MAb-based | 56 (0.9) | <5 (<0.4) | 54 (1.1) |

Abbreviations: IMiD, immunomodulatory drug; MAb, monoclonal antibody; PI, proteasome inhibitor; SCT, stem cell transplant.

**Supplementary Table S3.** Patient and disease characteristics among patients with MM who received SCT in line 1, by post-SCT consolidation and/or post-SCT maintenance therapy receipt

| **Characteristic,  *n* (%) unless otherwise noted** | **SCT group (*N* = 1127)** | | | |
| --- | --- | --- | --- | --- |
|  | **Post-SCT consolidation  (*n* = 80)** | **No post-SCT consolidation  (*n* = 1047)** | **Post-SCT maintenance**  **(*n* = 724)** | **No post-SCT maintenance**  **(*n* = 403)** |
| **Age at diagnosis (years)** |  |  |  |  |
| Median (IQR) | 63 (56 - 68) | 63 (57 - 69) | 63 (57 - 68) | 63 (56 - 69) |
| **Age at diagnosis (categorical years)** |  |  |  |  |
| <65 | 41 (51.25%) | 576 (55.01%) | 401 (55.39%) | 216 (53.6%) |
| 65 - <75 | 36 (45.0%) | 435 (41.55%) | 304 (41.99%) | 167 (41.44%) |
| ≥75 | 3 (3.75%) | 36 (3.44%) | 19 (2.62%) | 20 (4.96%) |
| **Age at start of LOT (years)** |  |  |  |  |
| Median (IQR) | 63 (56 - 68) | 63 (57 - 69) | 63 (57 - 68) | 63 (56 - 69) |
| **Age at start of LOT (categorical years)** |  |  |  |  |
| <65 | 41 (51.25%) | 572 (54.63%) | 399 (55.11%) | 214 (53.1%) |
| 65 - <75 | 36 (45.0%) | 438 (41.83%) | 306 (42.27%) | 168 (41.69%) |
| >=75 | 3 (3.75%) | 37 (3.53%) | 19 (2.62%) | 21 (5.21%) |
| **Sex** |  |  |  |  |
| Female | 33 (41.25%) | 463 (44.22%) | 317 (43.78%) | 179 (44.42%) |
| Male | 47 (58.75%) | 584 (55.78%) | 407 (56.22%) | 224 (55.58%) |
| **Race** |  |  |  |  |
| Non-Hispanic Black/African-American | 11 (13.75%) | 156 (14.9%) | 104 (14.36%) | 63 (15.63%) |
| Non-Hispanic White | 61 (76.25%) | 651 (62.18%) | 459 (63.4%) | 253 (62.78%) |
| Hispanic or Latinx | 4 (5.0%) | 79 (7.55%) | 59 (8.15%) | 24 (5.96%) |
| Other | 2 (2.5%) | 75 (7.16%) | 51 (7.04%) | 26 (6.45%) |
| Missing | 2 (2.5%) | 86 (8.21%) | 51 (7.04%) | 37 (9.18%) |
| **Region of residence** |  |  |  |  |
| Northeast | 12 (15.0%) | 160 (15.28%) | 104 (14.36%) | 68 (16.87%) |
| Midwest | 5 (6.25%) | 156 (14.9%) | 110 (15.19%) | 51 (12.66%) |
| South | 24 (30.0%) | 407 (38.87%) | 289 (39.92%) | 142 (35.24%) |
| West | 2 (2.5%) | 130 (12.42%) | 100 (13.81%) | 32 (7.94%) |
| Other/Missing | 37 (46.25%) | 194 (18.53%) | 121 (16.71%) | 110 (27.3%) |
| **Practice type** |  |  |  |  |
| Academic | 37 (46.25%) | 157 (15.0%) | 100 (13.81%) | 94 (23.33%) |
| Community | 43 (53.75%) | 871 (83.19%) | 615 (84.94%) | 299 (74.19%) |
| Academic+Community | 0 (0.00%) | 19 (1.81%) | 9 (1.24%) | 10 (2.48%) |
| **M-protein type** |  |  |  |  |
| IgG | 50 (62.5%) | 621 (59.31%) | 435 (60.08%) | 236 (58.56%) |
| IgA | 20 (25.0%) | 222 (21.2%) | 148 (20.44%) | 94 (23.33%) |
| Light chain | 10 (12.5%) | 173 (16.52%) | 121 (16.71%) | 62 (15.38%) |
| Other |  | 18 (1.72%) | 12 (1.66%) | 6 (1.49%) |
| Missing |  | 13 (1.24%) | 8 (1.1%) | 5 (1.24%) |
| **ISS stage at diagnosis** |  |  |  |  |
| Stage I | 29 (36.25%) | 341 (32.57%) | 237 (32.73%) | 133 (33.0%) |
| Stage II | 27 (33.75%) | 230 (21.97%) | 154 (21.27%) | 103 (25.56%) |
| Stage III | 14 (17.5%) | 193 (18.43%) | 127 (17.54%) | 80 (19.85%) |
| Missing | 10 (12.5%) | 283 (27.03%) | 206 (28.45%) | 87 (21.59%) |
| **Cytogenetic risk (assessed at any time)** |  |  |  |  |
| High risk^a^ | 18 (22.5%) | 217 (20.73%) | 142 (19.61%) | 93 (23.08%) |
| Standard risk | 16 (20.0%) | 282 (26.93%) | 192 (26.52%) | 106 (26.3%) |
| Missing | 46 (57.5%) | 548 (52.34%) | 390 (53.87%) | 204 (50.62%) |
| **1q21+**^b^ **(assessed at any time)** |  |  |  |  |
| Present | 21 (26.25%) | 243 (23.21%) | 164 (22.65%) | 100 (24.81%) |
| Absent | 24 (30.0%) | 398 (38.01%) | 280 (38.67%) | 142 (35.24%) |
| Missing | 35 (43.75%) | 406 (38.78%) | 280 (38.67%) | 161 (39.95%) |
| **eGFR**^c^ **(mL/min/1.73 m^2^) at start of LOT** |  |  |  |  |
| <60 | 10 (12.5%) | 264 (25.21%) | 190 (26.24%) | 84 (20.84%) |
| ≥60 | 42 (52.5%) | 519 (49.57%) | 369 (50.97%) | 192 (47.64%) |
| Missing | 28 (35.0%) | 264 (25.21%) | 165 (22.79%) | 127 (31.51%) |
| **ECOG PS at start of LOT** |  |  |  |  |
| 0 | 17 (21.25%) | 311 (29.7%) | 207 (28.59%) | 121 (30.02%) |
| 1 | 9 (11.25%) | 265 (25.31%) | 180 (24.86%) | 94 (23.33%) |
| ≥2 | 8 (10.0%) | 77 (7.35%) | 54 (7.46%) | 31 (7.69%) |
| Missing | 46 (57.5%) | 394 (37.63%) | 283 (39.09%) | 157 (38.96%) |
| **Year of LOT start** |  |  |  |  |
| 2016 | 15 (18.75%) | 188 (17.96%) | 137 (18.92%) | 66 (16.38%) |
| 2017 | 13 (16.25%) | 202 (19.29%) | 153 (21.13%) | 62 (15.38%) |
| 2018 | 18 (22.5%) | 209 (19.96%) | 155 (21.41%) | 72 (17.87%) |
| 2019 | 16 (20.0%) | 203 (19.39%) | 144 (19.89%) | 75 (18.61%) |
| 2020 | 16 (20.0%) | 178 (17.0%) | 109 (15.06%) | 85 (21.09%) |
| 2021 | 2 (2.5%) | 67 (6.4%) | 26 (3.59%) | 43 (10.67%) |
| **Time from MM diagnosis to LOT start (months)** |  |  |  |  |
| Median (IQR) | 1.06 (0.65 - 1.55) | 1.06 (0.68 - 1.48) | 1.06 (0.71 - 1.45) | 1.06 (0.65 - 1.55) |

Abbreviations: ECOG, Eastern Cooperative Oncology Group; EGFR, estimated glomerular filtration rate; Ig, immunoglobulin; IQR, interquartile range; ISS, International Staging System; LOT, line of therapy; MDRD, Modification of Diet in Renal Disease; MM, multiple myeloma; PS, performance status; SCT, stem cell transplant.

^a^High-risk cytogenetics were defined as the presence of ≥1 of del(17p), t(4;14), or t(14;16).

^b^1q21+ was defined as gain (3 copies) or amplification (≥4 copies) of 1q21.

^c^Assessed using the MDRD equation.

**Supplementary Table S4.** Line 1 MM treatment sequence among patients who received SCT in line 1 of therapy

| **Line 1 treatment sequencing, *n* (%)** | **SCT group (*N* = 1127)** |
| --- | --- |
| Primary: PI+IMiD --> Transplant: Yes --> Maintenance: IMiD | 446 (39.57%) |
| Primary: PI+IMiD --> Transplant: Yes | 216 (19.17%) |
| Primary: PI+IMiD --> Transplant: Yes --> Maintenance: PI | 79 (7.01%) |
| Primary: Chemo --> Transplant: Yes --> Maintenance: IMiD | 53 (4.7%) |
| Primary: Chemo --> Transplant: Yes | 43 (3.82%) |
| Primary: IMiD --> Transplant: Yes | 42 (3.73%) |
| Primary: PI+IMiD --> Transplant: Yes --> Consolidation: PI+IMiD | 40 (3.55%) |
| Primary: IMiD --> Transplant: Yes --> Consolidation: IMiD | 37 (3.28%) |
| Primary: IMiD --> Transplant: Yes --> Maintenance: IMiD | 36 (3.19%) |
| Primary: PI+IMiD --> Transplant: Yes --> Maintenance: PI+IMiD | 32 (2.84%) |
| Primary: Chemo --> Transplant: Yes --> Maintenance: PI | 26 (2.31%) |
| Primary: MAb --> Transplant: Yes | 18 (1.6%) |
| Primary: MAb --> Transplant: Yes --> Maintenance: IMiD | 11 (0.98%) |
| Primary: MAb --> Transplant: Yes --> Maintenance: MAb | 10 (0.89%) |
| Primary: Chemo --> Maintenance: IMiD --> Transplant: Yes --> Maintenance: IMiD | 6 (0.53%) |
| Primary: Chemo --> Transplant: Yes --> Maintenance: PI+IMiD | 6 (0.53%) |
| Primary: PI --> Transplant: Yes --> Maintenance: IMiD | 5 (0.44%) |
| Primary: PI --> Transplant: Yes | 4 (0.35%) |
| Primary: PI --> Transplant: Yes --> Maintenance: PI | 4 (0.35%) |
| Primary: IMiD --> Transplant: Yes --> Maintenance: PI | 3 (0.27%) |
| Primary: MAb --> Transplant: Yes --> Maintenance: PI+IMiD | 2 (0.18%) |
| Primary: PI --> Transplant: Yes --> Consolidation: PI | 2 (0.18%) |
| Primary: PI+IMiD --> Maintenance: PI --> Transplant: Yes --> Maintenance: PI | 1 (0.09%) |
| Primary: Chemo --> Transplant: Yes --> Maintenance: MAb | 1 (0.09%) |
| Primary: MAb --> Transplant: Yes --> Maintenance: PI | 1 (0.09%) |
| Primary: MAb --> Transplant: Yes --> Consolidation: MAb | 1 (0.09%) |
| Primary: IMiD --> Maintenance: IMiD --> Transplant: Yes | 1 (0.09%) |
| Primary: IMiD --> Maintenance: PI --> Transplant: Yes | 1 (0.09%) |

Abbreviations: Chemo, chemotherapy; IMiD, immunomodulatory drug; MAb, monoclonal antibody; MM, multiple myeloma; PI, proteasome inhibitor; SCT, stem cell transplant.

**Supplementary Table S5.** Real-world PFS from start of initial therapy, by SCT receipt in line 1 (all subgroups)

|  |  | **rwPFS Events** | |  |
| --- | --- | --- | --- | --- |
| **Subgroup** | **Person years at risk** | **RW progression, *n* (%)** | **Deaths, *n* (%)** | **MV-adjusted HR (95%CI)** |
| **Overall** | 8129.18 | 1619 (32.05%) | 945 (18.71%) | -- |
| No SCT | 6293.82 | 1405 (33.95%) | 886 (21.41%) | (Ref) |
| SCT | 1835.36 | 214 (23.41%) | 59 (6.46%) | 0.49 (0.43-0.57) |
| **Age at start of 1L** |  |  |  |  |
| **<65** | -- | -- | -- | -- |
| No SCT | 1743.8 | 341 (31.84%) | 143 (13.35%) | (Ref) |
| SCT | 990.43 | 117 (23.68%) | 30 (6.07%) | 0.52 (0.42-0.63) |
| **65 - <75** | -- | -- | -- | -- |
| No SCT | 2193.54 | 461 (33.82%) | 259 (19.0%) | (Ref) |
| SCT | 796.48 | 89 (22.76%) | 29 (7.42%) | 0.44 (0.36-0.54) |
| **≥75** | -- | -- | -- | -- |
| No SCT | 2356.48 | 603 (35.39%) | 484 (28.4%) | (Ref) |
| SCT | 48.44 | 8 (27.59%) | 0 (0.0%) | 0.40 (0.20-0.81) |
| **Sex** |  |  |  |  |
| **Female** | -- | -- | -- | -- |
| No SCT | 2899.58 | 666 (34.81%) | 365 (19.08%) | (Ref) |
| SCT | 855.43 | 98 (24.26%) | 28 (6.93%) | 0.50 (0.41-0.61) |
| **Male** | -- | -- | -- | -- |
| No SCT | 3394.24 | 739 (33.21%) | 521 (23.42%) | (Ref) |
| SCT | 979.93 | 116 (22.75%) | 31 (6.08%) | 0.49 (0.40-0.58) |
| **Race/Ethnicity** |  |  |  |  |
| **NH AA/Black** | -- | -- | -- | -- |
| No SCT | 1156.87 | 244 (32.62%) | 146 (19.52%) | (Ref) |
| SCT | 281.29 | 37 (25.69%) | 7 (4.86%) | 0.55 (0.39-0.77) |
| **Hispanic (Any race)** | -- | -- | -- | -- |
| No SCT | 410.96 | 99 (39.44%) | 33 (13.15%) | (Ref) |
| SCT | 144.57 | 20 (28.57%) | 2 (2.86%) | 0.39 (0.23-0.64) |
| **NH White** | -- | -- | -- | -- |
| No SCT | 3637.34 | 787 (34.52%) | 533 (23.38%) | (Ref) |
| SCT | 1196.24 | 125 (21.7%) | 47 (8.16%) | 0.50 (0.42-0.59) |
| **ISS stage at diagnosis** |  |  |  |  |
| **Stage I** | -- | -- | -- | -- |
| No SCT | 1448.94 | 249 (31.52%) | 90 (11.39%) | (Ref) |
| SCT | 658.48 | 51 (16.67%) | 13 (4.25%) | 0.40 (0.30-0.54) |
| **Stage II** | -- | -- | -- | -- |
| No SCT | 1294.19 | 303 (35.52%) | 173 (20.28%) | (Ref) |
| SCT | 399.28 | 50 (23.7%) | 18 (8.53%) | 0.48 (0.36-0.63) |
| **Stage III** | -- | -- | -- | -- |
| No SCT | 1227.92 | 322 (36.51%) | 236 (26.76%) | (Ref) |
| SCT | 303.37 | 53 (32.32%) | 17 (10.37%) | 0.57 (0.43-0.74) |
| **Cytogenetic risk** |  |  |  |  |
| **Standard risk** | -- | -- | -- | -- |
| No SCT | 1821.25 | 398 (32.7%) | 238 (19.56%) | (Ref) |
| SCT | 502.95 | 41 (16.87%) | 13 (5.35%) | 0.38 (0.28-0.51) |
| **High risk^a^** | -- | -- | -- | -- |
| No SCT | 849.63 | 242 (40.2%) | 146 (24.25%) | (Ref) |
| SCT | 363.17 | 69 (35.2%) | 19 (9.69%) | 0.46 (0.36-0.60) |
| **del(17p)** |  |  |  |  |
| **del(17p) absent** | -- | -- | -- | -- |
| No SCT | 3929.73 | 821 (33.44%) | 481 (19.59%) | (Ref) |
| SCT | 1240.89 | 130 (21.56%) | 35 (5.8%) | 0.46 (0.39-0.55) |
| **del(17p) present** | -- | -- | -- | -- |
| No SCT | 539.01 | 156 (41.6%) | 90 (24.0%) | (Ref) |
| SCT | 202.36 | 40 (36.36%) | 11 (10.0%) | 0.55 (0.39-0.78) |
| **1q21+^b^** |  |  |  |  |
| **Absent** | -- | -- | -- | -- |
| No SCT | 2436.61 | 473 (31.83%) | 294 (19.78%) | (Ref) |
| SCT | 740.26 | 68 (19.71%) | 29 (8.41%) | 0.49 (0.39-0.62) |
| **Present** | -- | -- | -- | -- |
| No SCT | 1215.92 | 324 (38.12%) | 196 (23.06%) | (Ref) |
| SCT | 426.42 | 72 (32.0%) | 9 (4.0%) | 0.45 (0.34-0.58) |
| **eGFR^c^ (mL/min/1.73 m^2^)** |  |  |  |  |
| **≥60** | -- | -- | -- | -- |
| No SCT | 2631.59 | 528 (33.19%) | 283 (17.79%) | (Ref) |
| SCT | 964.45 | 102 (21.98%) | 30 (6.47%) | 0.47 (0.38-0.57) |
| **<60** | -- | -- | -- | -- |
| No SCT | 2175.18 | 531 (34.57%) | 415 (27.02%) | (Ref) |
| SCT | 462.52 | 56 (24.67%) | 16 (7.05%) | 0.45 (0.35-0.58) |
| **Primary line 1 treatment** |  |  |  |  |
| **VRd** | -- | -- | -- | -- |
| No SCT | 3221.41 | 679 (33.81%) | 329 (16.38%) | (Ref) |
| SCT | 1250.15 | 137 (22.99%) | 37 (6.21%) | 0.47 (0.39-0.56) |
| **CyBorD** | -- | -- | -- | -- |
| No SCT | 708.99 | 160 (35.01%) | 132 (28.88%) | (Ref) |
| SCT | 121.81 | 18 (30.0%) | 4 (6.67%) | 0.55 (0.35-0.87) |

Abbreviations: 1L, first line; AA, African American; CI, confidence interval; CyBorD, cyclophosphamide/bortezomib/dexamethasone; eGFR, estimated glomerular filtration rate; HR, hazard ratio; ISS, International Staging System; MDRD, Modification of Diet in Renal Disease; MM, multiple myeloma; MV, multivariable; NH, non-Hispanic; PFS, progression-free survival; rwPFS, real-world progression-free survival; RW, real-world; SCT, stem cell transplant; VRd, bortezomib/lenalidomide/
dexamethasone.

^a^High-risk cytogenetics were defined as the presence of ≥1 of del(17p), t(4;14), or t(14;16).

^b^1q21+ was defined as gain (3 copies) or amplification (≥4 copies) of 1q21.

^c^Assessed using the MDRD equation.

**Supplementary Table S6.** Real-world OS from start of initial therapy, by SCT receipt in line 1 (all subgroups)

| **Subgroup** | **Person years at risk** | **Deaths, *n* (%)** | **MV-adjusted HR (95%CI)** |
| --- | --- | --- | --- |
| **Overall** | 12634.04 | 1824 (30.42%) | -- |
| No SCT | 9833.89 | 1684 (34.59%) | (Ref) |
| SCT | 2800.15 | 140 (12.42%) | 0.47 (0.39-0.56) |
| **Age at start of 1L** |  |  |  |
| **<65** | -- | -- | -- |
| No SCT | 2761.42 | 284 (22.9%) | (Ref) |
| SCT | 1537.16 | 72 (11.75%) | 0.45 (0.35-0.60) |
| **65 - <75** | -- | -- | -- |
| No SCT | 3449.97 | 514 (31.97%) | (Ref) |
| SCT | 1177.6 | 63 (13.29%) | 0.43 (0.33-0.56) |
| **≥75** | -- | -- | -- |
| No SCT | 3622.5 | 886 (43.84%) | (Ref) |
| SCT | 85.4 | 5 (12.5%) | 0.31 (0.13-0.75) |
| **Sex** |  |  |  |
| **Female** | -- | -- | -- |
| No SCT | 4567.35 | 738 (32.98%) | (Ref) |
| SCT | 1252.65 | 62 (12.5%) | 0.48 (0.36-0.63) |
| **Male** | -- | -- | -- |
| No SCT | 5266.54 | 946 (35.96%) | (Ref) |
| SCT | 1547.5 | 78 (12.36%) | 0.45 (0.35-0.58) |
| **Race/Ethnicity** |  |  |  |
| **NH AA/Black** | -- | -- | -- |
| No SCT | 1746.16 | 280 (33.06%) | (Ref) |
| SCT | 410.03 | 21 (12.57%) | 0.52 (0.33-0.84) |
| **Hispanic (Any race)** | -- | -- | -- |
| No SCT | 679.09 | 79 (24.61%) | (Ref) |
| SCT | 205.76 | 7 (8.43%) | 0.28 (0.12-0.64) |
| **NH White** | -- | -- | -- |
| No SCT | 5635.98 | 993 (37.69%) | (Ref) |
| SCT | 1849.02 | 99 (13.9%) | 0.49 (0.39-0.61) |
| **ISS stage at diagnosis** |  |  |  |
| **Stage I** | -- | -- | -- |
| No SCT | 2138.45 | 168 (19.16%) | (Ref) |
| SCT | 957.28 | 29 (7.84%) | 0.41 (0.27-0.63) |
| **Stage II** | -- | -- | -- |
| No SCT | 2016.34 | 299 (31.41%) | (Ref) |
| SCT | 609.94 | 35 (13.62%) | 0.54 (0.37-0.79) |
| **Stage III** | -- | -- | -- |
| No SCT | 1949.74 | 454 (43.44%) | (Ref) |
| SCT | 486.45 | 45 (21.74%) | 0.49 (0.35-0.68) |
| **Cytogenetic risk** |  |  |  |
| **Standard risk** | -- | -- | -- |
| No SCT | 2762.44 | 435 (30.74%) | (Ref) |
| SCT | 755.32 | 23 (7.72%) | 0.30 (0.19-0.47) |
| **High risk^a^** | -- | -- | -- |
| No SCT | 1354.94 | 307 (43.12%) | (Ref) |
| SCT | 538.62 | 54 (22.98%) | 0.52 (0.38-0.72) |
| **del(17p)** |  |  |  |
| **del(17p) absent** | -- | -- | -- |
| No SCT | 6067.81 | 874 (30.88%) | (Ref) |
| SCT | 1854.89 | 79 (10.78%) | 0.45 (0.35-0.57) |
| **del(17p) present** | -- | -- | -- |
| No SCT | 880.85 | 209 (46.65%) | (Ref) |
| SCT | 308.67 | 35 (26.32%) | 0.64 (0.43-0.96) |
| **1q21+^b^** |  |  |  |
| **Absent** | -- | -- | -- |
| No SCT | 3659.73 | 525 (30.92%) | (Ref) |
| SCT | 1090.11 | 54 (12.8%) | 0.54 (0.40-0.74) |
| **Present** | -- | -- | -- |
| No SCT | 1904.69 | 392 (39.52%) | (Ref) |
| SCT | 628.25 | 38 (14.39%) | 0.40 (0.28-0.57) |
| **eGFR^c^ (mL/min/1.73 m^2^)** |  |  |  |
| **≥60** | -- | -- | -- |
| No SCT | 3986.48 | 516 (28.96%) | (Ref) |
| SCT | 1434.45 | 69 (12.3%) | 0.51 (0.39-0.68) |
| **<60** | -- | -- | -- |
| No SCT | 3378.01 | 755 (42.34%) | (Ref) |
| SCT | 677.26 | 36 (13.14%) | 0.38 (0.26-0.53) |
| **Primary line 1 treatment** |  |  |  |
| **VRd** | -- | -- | -- |
| No SCT | 4859.81 | 627 (28.32%) | (Ref) |
| SCT | 1873.36 | 94 (12.88%) | 0.54 (0.43-0.68) |
| **CyBorD** | -- | -- | -- |
| No SCT | 1146.09 | 235 (42.57%) | (Ref) |
| SCT | 213.25 | 13 (17.11%) | 0.40 (0.22-0.72) |

Abbreviations: 1L, first line; AA, African American; CI, confidence interval; CyBorD, cyclophosphamide/bortezomib/dexamethasone; eGFR, estimated glomerular filtration rate; HR, hazard ratio; ISS, International Staging System; MDRD, Modification of Diet in Renal Disease; MM, multiple myeloma; MV, multivariable; NH, non-Hispanic; OS, overall survival; SCT, stem cell transplant; VRd, bortezomib/lenalidomide/dexamethasone.

^a^High-risk cytogenetics were defined as the presence of ≥1 of del(17p), t(4;14), or t(14;16).

^b^1q21+ was defined as gain (3 copies) or amplification (≥4 copies) of 1q21.

^c^Assessed using the MDRD equation.

**Supplemental Figure S1**. Attrition diagram


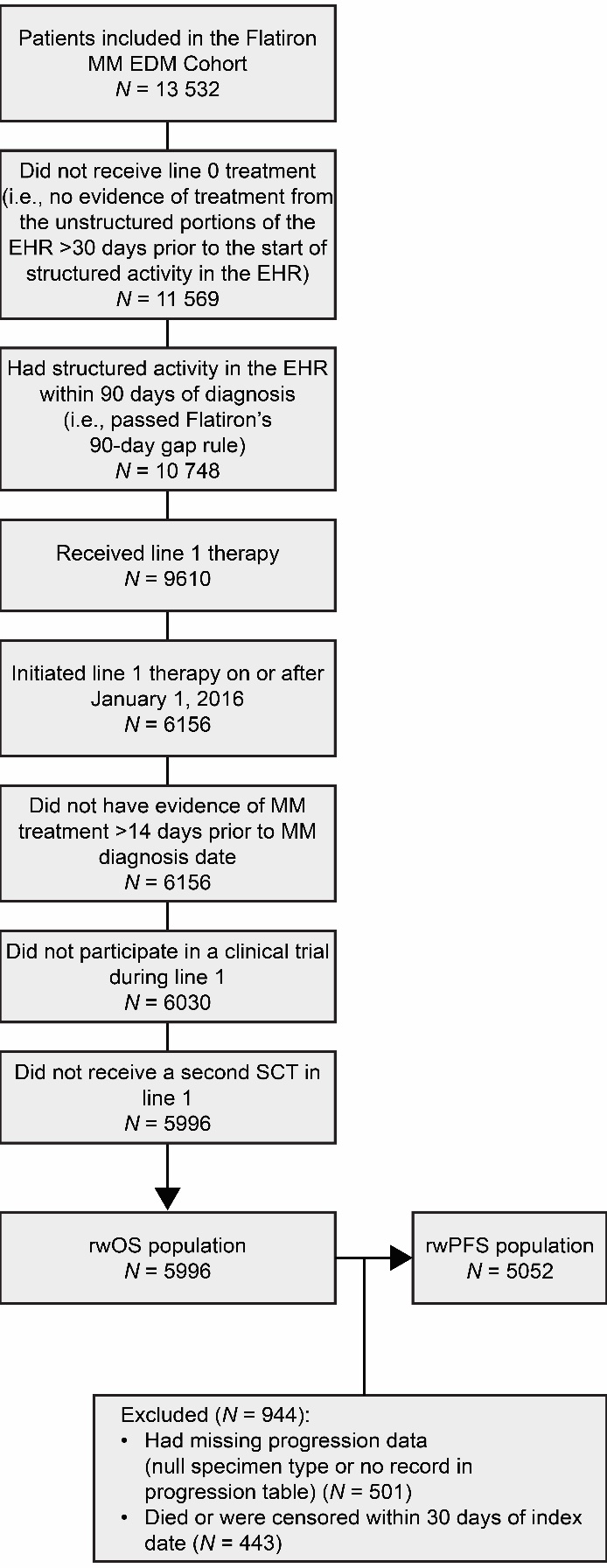

Supplement: Supplementary file 1 — Supporting Information [file JHA2-4-984-s001.docx]
